# Hard to Avoid but Difficult to Sustain: Scotland's Innovative Health Tax on Large Retailers Selling Tobacco and Alcohol

**DOI:** 10.1111/1468-0009.12200

**Published:** 2016-12-19

**Authors:** MARK HELLOWELL, KATHERINE E. SMITH, ALEXANDRA WRIGHT

**Affiliations:** ^1^ School of Social and Political ScienceUniversity of Edinburgh

**Keywords:** alcohol, tobacco, taxes, retailers/supermarkets

## Abstract

**Policy Points:**

- The Scottish government's Public Health Supplement (a levy on large retailers selling alcohol and tobacco) was an innovative tax that was successful in generating predictable revenues but failed to stimulate substantial behavioral change among retailers (eg, decisions to stop selling alcohol or tobacco).
- A tax may be considered a “health” tax on the basis that it raises revenue for health spending and/or is intended to achieve health aims (eg, behavioral change among suppliers or consumers of health‐damaging products), but there is likely to be tension between these goals in terms of policy design.
- Although framing a tax as a “health” measure may increase public support, where the substantive health content is limited, or questionable, such a measure may be difficult to sustain in the face of industry criticism and a lack of competing support from health interests.

**Context:**

In the context of increasing health spending and a constrained budget, the Scottish government levied a new “health” tax on large retailers selling alcohol and tobacco in April 2012. This innovative tax, the Public Health Supplement, had the potential to finance additional health spending while discouraging retailers from selling tobacco. We present a case study of the levy; examine how it evolved over time and what impacts it had; explore why, in 2015, the government decided to discontinue the policy; and consider how this experience might inform future strategies for addressing tobacco and alcohol harms via taxes on retailers.

**Methods:**

We employed 3 data sources: (1) policy documents (both documents in the public domain and documents obtained via Freedom of Information requests), (2) media coverage of the debates surrounding the Public Health Supplement, and (3) key informant interviews. We analyzed these data collectively, in chronological order, triangulating between sources.

**Findings:**

When the Supplement was announced in 2011, a clear health rationale was advanced. However, the policy, as subsequently implemented, was not designed to elicit a behavioral response from retailers in terms of alcohol or tobacco sales. It was successful in generating a predictable revenue stream, but there was no evidence that this was earmarked for health. Hence, the substantive health content of the policy was questionable, a fact that was highlighted by industry opponents of the tax, while there was also a lack of competing support from health interests. The industry's campaign was influential in the government's subsequent decision to reduce the rate of the tax and restrict its duration to 3 years.

**Conclusions:**

A tax may be considered a “health” tax on the basis that it raises revenue for health spending and/or that it helps achieve health aims (eg, behavioral change), but there may be tension between these goals in policy design. Framing a tax as a health measure may increase public support in the short term, but this may not be sustained if such framing is insufficiently justified.

In september 2011, the scottish government announced a new health tax, the Public Health Supplement (henceforth the Supplement). This tax, which was in place between April 2012 and March 2015, was levied on retail premises in Scotland selling *both* alcohol and tobacco that had a ratable value of over £300,000. It was an innovative tax that, in contrast to traditional tobacco and alcohol excise taxes, targeted large supermarkets selling alcohol and tobacco products. As far as we are aware, this was the first tax of its kind.

The Supplement was first announced on September 21, 2011, as part of the Spending Review 2011 and Draft Budget 2012‐13.^1^ In that document, the aims were recorded as addressing the health and social problems associated with alcohol and tobacco use and generating income for “preventive spending.” Three key factors appear to explain the Scottish government's interest in developing this kind of tax. First, within the United Kingdom, Scotland has a relatively high burden of smoking‐ and alcohol‐related problems. In economic terms, alcohol misuse is estimated to cost £3.6 billion a year in Scotland,^2^ while tobacco is estimated to cost £1.1 billion a year.^3^ In health terms, recent data suggest that alcohol contributes to approximately 1,000 deaths a year in Scotland,^4^ while around 13,000 deaths a year are attributable to smoking.^5^ Second, although responsibility for health policy in Scotland has been devolved since 1998 (following the creation of a new Scottish Parliament), the Scottish government's ability to raise revenue via taxation was limited until further devolution of tax raising powers in 2016.^6^ The parameters of the Scottish government's spending plans were set by UK‐level decisions on public spending, a constraint that had become increasingly salient in the context of Scottish government criticism of the UK government's austerity agenda cuts.^7^ Third, following its “first mover” status in areas such as smoke‐free regulation^6^ and legislating for a minimum unit price for alcohol,^8^ the Scottish government had developed a reputation as something of a health policy leader in the United Kingdom.^6^, ^9^

The Scottish government initially predicted that the levy would raise £110 million over 3 years. This estimate was subsequently revised down to £95 million over 3 years. In 2015, for reasons that were not made clear, the Scottish government decided not to continue the levy into the next 3‐year budgeting period. No formal evaluation of the Supplement has been undertaken. Indeed, as far as we are aware, this paper presents the first analysis of this notable measure, using a case study approach to examine the process through which the policy was developed, its evolution over the period in which it was implemented, the resulting outcomes, and the subsequent decision to discontinue the policy. Drawing on this case study, we consider how these experiences can inform future strategies for policymakers and advocates interested in addressing tobacco and harmful alcohol through new taxes on retailers.

## Methods

We employed a qualitative case study approach, drawing on 3 key data sources (relevant documents, media coverage and key informant interviews), triangulating the results across these sources. For the documentary analysis, we began by searching the Scottish government and Scottish Parliament websites for documents concerning the Supplement. The most relevant sources of information from these searches were downloaded and saved for analysis. We also submitted a Freedom of Information request to the Scottish government to access information on the nature and extent of government–industry interactions in the period between the Supplement being announced in September 2011 and the statement of the finalized policy in February 2012. In addition, we searched the Internet more widely to try to identify documents relating to the Supplement produced by interested stakeholders, focusing on those produced by large retailers. We also searched for documents cited in media reports that appeared to be of interest and asked all interviewees to let us know about any potentially useful documents. We analyzed the resulting documents collectively, in chronological order, to ascertain which actors were involved in discussions about the Supplement, to understand the details of this policy's development, and to assess how discussions about the Supplement changed over time. Box 1 identifies the key documents included in our analysis.

Box 1 **Key Documents Included in Our Analysis**

- Scottish Spending Review 2011 and Draft Budget 2012‐13
- Budget Speech by Finance Minister John Swinney, 2012
- Oral and Written Answers to Oral and Written Questions in the Scottish Parliament
- Information received via Freedom of Information request, including emails and letters between the Scottish government and Tesco, the Confederation of British Industry (CBI Scotland), and the Scottish Retail Consortium regarding the Public Health Supplement, along with minutes of related meetings
- Information related to meetings between the Scottish government and other policy actors in the public domain
- Reports of Scottish Parliament scrutiny of the 2011 Spending Review and 2012‐13 Draft Budget
- Oral and Written Evidence to Scottish Parliamentary Committee inquiries and
- Regulations and Executive Note for the Public Health Supplement

We were not able to obtain copies of all of the documents we identified as relevant, which was a limitation. One important document we were not able to obtain was a report produced by the Centre for Economics and Business Research (CEBR), a consultancy organization, on behalf of Asda, one of the large supermarket chains affected by the Supplement. The report was widely referred to in media coverage of the Supplement and was drawn on by large retailers in their efforts to mobilize against the Supplement. In our efforts to obtain a copy of the report, we submitted requests to both Asda and CEBR, asked interviewees whom we knew had seen the report, and queried journalists who had written articles that cited the report, but none of these routes proved successful. The media coverage of this report provided us with information about the report's contents but, without the full report, we were not able to analyze specific claims made in this document in any detail.

At the same time, we undertook searches for relevant media articles in two databases: Factiva and ProQuest (specifically the ABI/INFORM Complete and ISSB databases). These databases were selected (following discussions with a qualified librarian) for their breadth of media coverage, both geographically and in terms of the types of media suggested (ie, including trade journals and other specialist media). After undertaking an initial analysis, we developed search strings (outlined in Box 2) that included the various phrases we identified as being used to describe the Supplement in media discussions and combined these with “Scotland,” our geographical focus, which helped exclude nonrelevant articles.

Box 2 **Media Analysis Search Strings**

Dates of Search: July 10, 2015, to July 15, 2015

**Database**: Factiva

**Search String**: (“public health levy” OR “public health tax” OR “public health supplement” OR “large retailer's tax” OR “large retailer's levy” OR “Tesco tax” OR “supermarket tax”) AND Scotland

**Filter**: 2010‐2015

**Database**: ProQuest (ABI/INFORM Complete and ISSB)

**Search string**: (“public health levy” OR “public health tax” OR “public health supplement” OR “large retailer's levy” OR “supermarket tax” OR “Tesco tax”) AND Scotland

**Filter**: Exclude scholarly journals

The media searches were conducted in July 2015 (ie, several months after the Public Health Supplement was discontinued). The combined results were screened for relevance and the relevant articles (n = 151) were then analyzed thematically, using a data extraction table that focused on identifying (1) which actors were involved in debates about the Supplement, and (2) the main claims and arguments put forward about the Supplement.

Using the results of the documentary and media analysis, we then identified a list of potential interviewees. We subsequently asked all interviewees to identify other potentially relevant interviewees. In total, we approached 32 individuals with a request for an interview, of which only 9 agreed to participate. This is a relatively low response rate, but is reflective of the fact that, as shown below, the Supplement was conceived and designed by a small number of individuals working within the finance directorate of the Scottish government, and many of the actors we had anticipated would have a view on the Supplement did not feel that they had engaged with the policy sufficiently to be able to provide a useful interview. (This was the reason given for declining our interview request by individuals at NHS Health Scotland, local authorities, and health‐focused nongovernmental organizations [NGOs], as well as several opposition members of the Scottish Parliament [MSPs]). Indeed, 2 of the health‐focused NGOs we contacted stated that they did not view the Supplement as a public health measure (something we discuss in more detail later in the paper). Others—especially the large retailers, Scottish National Party (SNP) MSPs, ministers, and special advisers we approached—tended either not to reply or to say that they did not have time for an interview on this topic. A summary of the professional location of potential and actual interviewees is provided in Table 1.

**Table 1:** The Professional Locations of Individuals With Whom We Requested an Interview and Summary Response

Professional Location of Interviewees	Number of Interviewees Approached	Number of Interviewees Who Accepted
Finance Minister, John Swinney, and relevant special adviser	2	0
Individuals responsible for Scottish policy at 6 large supermarkets affected by Supplement	6	1 (plus email correspondence with 1 other)
Local government organizations	3	0
MSPs (from Conservative, Labour, Liberal Democrat, and SNP parties)	6	2 (both Labour Party; ie, opposition MSPs)
NHS Health Scotland (special health board with responsibility for public health in Scotland)	3	1
Nongovernmental organizations (with health focus)	4	1
Scottish government civil servant—health	2	1
Scottish government civil servant—finance/local government	2	1
Individuals working in the Scottish retail sector	3	2
Transnational tobacco company (the only one for which we identified any evidence of interest in policy debates around the supplement)	1	0
**Total**	**32**	**9**

Interviews were undertaken by one or both of the lead authors (wherever possible, we undertook interviews jointly but the limited availability of some interviewees meant that we had to conduct 4 of the interviews on an individual basis). All interviews were digitally recorded and professionally transcribed, with the written consent of interviewees. The one exception to this was the interview with an NHS Health Scotland staff member due to the extremely short interview length and the informant's lack of specific knowledge about the Supplement; summary notes were therefore deemed more appropriate. Interview transcripts were uploaded to NVivo, a qualitative data analysis software program, where they were thematically coded using a coding framework that reflected our primary interests (ie, to understand the rationale for implementing, and later discontinuing, the Public Health Supplement and to identify the key actors and arguments involved in debates about the measure).

To contextualize the specific findings of this project, we undertook 2 linked systematic searches for relevant literature concerning health taxes, focusing on (1) taxes intended to change the costs of supplying or consuming health‐damaging products and (2) taxes intended to be hypothecated for health‐related objectives. We discuss the results of these literature searches in more detail elsewhere.^10^, ^11^ In the context of the current paper, it is sufficient to note that we were unable to identify any similar taxes on retailers of tobacco and alcohol, suggesting that the Supplement represents an innovative health policy, and that this analysis is therefore an important opportunity to reflect on the lessons that might be learned from this novel form of taxation.

## Results

### How Was the Public Health Supplement Perceived by Key Stakeholders?

The stated aims of the Supplement when it was announced on September 21, 2011, were to address “the health and social problems associated with alcohol and tobacco use” and to generate income for preventive‐spending measures.^1^ Our media analysis shows that the policy was initially welcomed by public health NGOs, which tended to regard it as a means of reducing the supply of alcohol and tobacco while raising revenue for health spending:

*Scotland has again shown leadership in acting to curb the harm from this lethal and addictive product and to invest in our health*. —ASH Scotland^12^

In contrast, our interview data show that retail‐sector representatives viewed the Supplement as no more than a relabeled form of an earlier attempt to increase tax revenues from large retailers—a measure that had not contained any explicit public health content or framing:

*In the previous Parliament they had brought forward a proposal […] for a larger retailers levy […] but there was no linkage at all in name or otherwise to public health, booze, tobacco or anything like that. There were several different reasons given at the time. One was to tilt the balance in favor of smaller retailers, […] others it was about out‐of‐town retailers. […] And obviously ultimately they lost that vote. And then in subsequent Parliament when they got a majority, it miraculously became something to do with health*. —Interviewee from the retail sector

From this perspective, the Supplement was seen as no more than a means of raising additional revenue in order to address a financing gap in the Spending Review, which, as noted, was written in a context in which the block grant awarded to Scotland by the United Kingdom was being reduced, in real terms, for the first time since devolution.^13^ Our interview data show that, over time, the perception that the Supplement was largely a revenue‐raising measure and had no substantive public health content became dominant across all policy stakeholders.

In accordance with this interpretation, it is notable that health‐related actors were not included in discussions about the policy's development, even within the Scottish government. An interviewee from the Scottish government health directorate stated that the Supplement was primarily developed within the finance directorate. In addition, documents show that NHS Health Scotland—the national health agency tasked with improving health in the country—was not consulted in the development of the Supplement,^14^ and our attempts to interview individuals working at NHS Health Scotland confirmed this. Similarly, an employee of a public health NGO that we interviewed said they had not been consulted on the policy and had had no “advance warning” of it before its announcement in the Spending Review.

Perhaps as a consequence of this, other interviewees suggested that there was no strong lobby in favor of the Supplement, with one opposition MSP reflecting that this was surprising, “if it was a genuine health Supplement.”

### How Did Large Retailers Respond to the Supplement?

**Figure 1:**
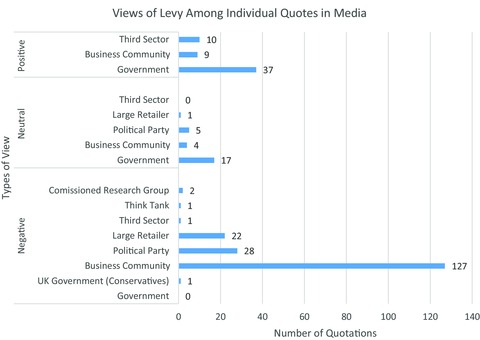
Comparing the Number of Positive, Negative, and Neutral Views on the Public Health Supplement by Individual Quotations Within Media Stories

The announcement of the Supplement elicited an intense and sustained response from large retailers that sell alcohol and tobacco (ie, Asda, the Co‐operative Group, Morrison's, Sainsbury's, and Tesco) and their representative bodies. Responses included (1) oral and written responses to Scottish Parliament committees; (2) direct lobbying of John Swinney, the then finance minister, and his officials, particularly during the period from the draft budget's publication on September 9, 2011, to the final budget speech on February 8, 2011; and (3) direct lobbying of the health directorate—even though, as outlined above, it was not a key player in developing the policy. The industry also engaged in a sustained campaign in the media, in which quotations that were critical of the Supplement were prominent (Figure 1).

**Figure 2:**
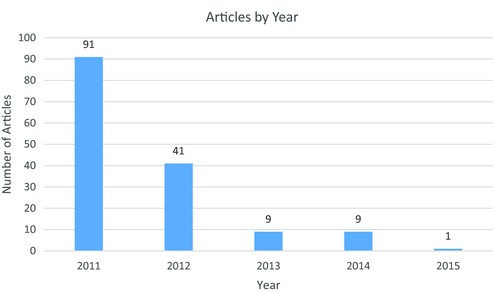
Number of News Articles Concerning the Public Health Supplement, by Year

As Figure 2 illustrates, there was a large increase in media coverage of the Supplement in the initial days and months following the announcement. The number of relevant media articles then decreased significantly over the next 4 years and, notably, did not peak in the run‐up to the announcement to discontinue the Supplement, suggesting that most of the public lobbying undertaken in relation to the Supplement occurred immediately after the measure was announced and before the details of the measure were confirmed. Between 2013 and 2015, the small number of media articles published appeared almost exclusively in newspapers, with only one article in a retail trade magazine published in 2013, which suggests that, although efforts to gain media attention were limited in this period, they remained outward facing.

Taken together, our data sources identify 8 distinct arguments employed against the Supplement, as summarized in Table 2. The frequency with which specific arguments were deployed, and their use across different sectors, varied. In the following subsections, we explain each of the arguments against the Supplement in detail.

**Table 2:** The Eight Distinct Arguments Used Against the Public Health Levy

Argument Against the Public Health Levy	No. of Times Argument Made in Media	Type of Interviewees Making Argument	Argument Evident in Documents?
1. Harmful economic impacts	55	Interviewees from the retail sector	Yes
2. Unfair targeting of part of the retail sector	31	Interviewees from the retail sector	Yes
3. Criticism of presentation as a health tax	20	All interviewees	Yes
4. Lack of impact assessment	18	Interviewees from the retail sector	Yes
5. Sets a worrying precedent	10	Interviewees from the retail sector	No
6. Lack of consultation	7	Interviewees from the retail sector	Yes
7. Increased prices for consumers	7	No interviewees made this argument	No
8. Not a legal measure	1	No interviewees made this argument	Yes

In a November 24, 2011, letter to John Swinney, obtained via a Freedom of Information request, CBI Scotland claimed the Supplement would “reduce rates of return and make it more expensive for retail to invest in Scotland than elsewhere in the United Kingdom or indeed abroad.”^15^ Asda commissioned CEBR to produce a report on the likely impacts of the Supplement, focusing on negative economic impacts. (It is perhaps worth noting that CEBR had previously been commissioned by the alcohol producer SABMiller to produce a report that critically assessed the economic modeling undertaken by academics at the University of Sheffield, which was being used to support Scottish government plans for minimum unit pricing for alcohol.^16^) The CEBR report focusing on the Supplement (which we have been unable to obtain, despite written requests to Asda and CEBR, among other organizations) was employed by the large retail sector in subsequent lobbying efforts, and its findings were summarized in some of the media coverage. From this coverage, it appears that the Supplement was framed in this report as an “economically irrational tool,” and estimates were put forward that the affected retailers would lose 8%‐10% of their profits.^17^

The interview data suggest that the predictions of negative economic impacts set out in the CEBR report were subsequently drawn on by large retailers and their representative groups in direct lobbying activities. One interviewee from a large retailer (not Asda) stated:

*You have to look at the effect [of the Supplement] on the profitability of particular stores, and profitability is the reason that investors decide where to put their money and where to invest, and the evidence [from the Asda‐commissioned CEBR report] suggested that the impact on store profitability was something like 10% of store operations, a store operation's profit, or 10% of the particular company's profitability across a group of stores. Now that is a massive impact compared with other places that people or companies could invest. And there were concerns about the impact, what message that sent to investors*.

Claiming that the Supplement would have negative economic impacts was also the most frequently employed argument against the policy in the media coverage. For example, as the above quotation hints, opponents argued that the Supplement would be a disincentive for investment by large retailers in Scotland, slowing their rate of expansion, and that it would harm their ability to sustain their contributions to the economy by providing jobs. Overall, the Supplement was positioned as directly counterproductive to the Scottish government's overarching economic goals. These goals were articulated in a foreward to the spending review, authored by then finance minister Swinney, as “accelerating economic recovery to create the jobs that our people need.”^1^

The lack of public health substance to the Supplement was also a major theme in the attempts of large retailers to lobby against it. In a November 8, 2011, letter to the Scottish government—which we obtained via a request made under the Freedom of Information Act—jointly written by the Scottish Retail Consortium (SRC), CBI Scotland, the Wine and Spirit Trade Association, and the Scottish Chambers of Commerce, these trade representatives argued:

*The link between the levy and preventative health measures is tenuous*. […] *There must be robust evidence that the levy will fund measurable, well‐defined and evidence‐based preventative health measures*.^18^

This was also a major theme in media coverage. For example:

*The Scottish government has a hole in its local authority budget and has chosen the retail sector to fill it, simply because supermarkets are profitable businesses. The public‐health justification for this levy is completely unfounded*. —Jane Bevis, SRC communications director, October 18, 2011^19^

The claims by opponents of the levy also highlighted the lack of a commitment to ring‐fencing the revenue raised through the levy for public health purposes:

*A true health measure would be properly evidence‐based, would not discriminate in this arbitrary way, and the revenue would be ring‐fenced for health purposes*. —SRC spokesperson, October 5, 2011^20^

Our analysis suggests that in addition to threats about investment and the economy, opponents also argued that the levy was discriminatory on the basis that it targeted only one part of one sector and that it could easily be extended to others:

*This levy still sets an alarming precedent by singling out one part of one sector, and businesses of all kinds will fear what future revenue‐raising schemes might be devised*. —Ian Shearer, SRC director, February 8, 2012^21^

There were 3 elements to this argument as presented by large retailers in the media coverage. One was to question the health basis of the Supplement's exclusive focus on larger retailers, given that many people purchase tobacco and alcohol products from smaller retailers. Another was to suggest that the Supplement potentially set a precedent for other sectors (an argument that appears to have been intended to quash any policy support from smaller retailers, as discussed in more detail below). A third was to claim that large retailers had a good reputation in the area of alcohol and tobacco sales, as well as participation in health‐related initiatives, and that the Supplement undermined these efforts. In the Scottish government itself, there was some sympathy with this latter argument. For example, the official we interviewed from the Scottish government health directorate indicated that the Supplement featured in public health–related conversations between the Scottish government and large retailers, with large retailers presenting the Supplement as simply displacing custom for tobacco and alcohol from large retailers to smaller retailers. Without any evaluation of the actual impacts on trade and consumption, this interviewee reflected that there had been no evidence‐base to assess this claim or enable the Scottish government to respond with a public health argument.

Efforts were also made to position large supermarkets as “vulnerable” entities that were being victimized by the levy and to suggest that this would lead to increased prices for consumers.^22^

There were also a set of arguments against the Supplement that related to the process via which the policy had been developed (arguments 4 and 6 in Table 2); because of its alleged economic effects, large retailers and their lobbyists argued, in the media, documents, and interviews (see Table 2) that the Supplement should have been subject to a Business Regulatory Impact Assessment (a tool to help policymakers consider the impacts of policy proposals on economic actors) and to far greater consultation. For example:

*Well there's a whole set of principles, core regulation principles. And clearly if you're going to do a major policy like that, then you should do some evidence beforehand, you should do some research, some assessment of what the costs and benefits would be. And there was no regulatory assessment at all, and the Scottish Government specifically said it wasn't going to do one, which completely contradicted all the guidelines about better regulation*. —Interviewee from the retail sector

The strong and frequent emphasis that business actors placed on “better regulation” guidelines and the need for a business impact assessment is interesting in the context of research demonstrating business (including tobacco company) involvement in shaping and promoting the better regulation agenda^23^ and impact assessment tools in the United Kingdom and the European Union.^24^

In addition, in the aforementioned November 8, 2011, letter from the SRC, CBI Scotland, the Wine and Spirit Trade Association, and the Scottish Chambers of Commerce to the Scottish government, the trade bodies argued that the Supplement was “a completely new form of taxation” for large retailers and business in general and required close scrutiny as such.^18^ In the weeks following the announcement of the Supplement, as it became clear that the Scottish government did not intend to carry out a Business Regulatory Impact Assessment on the Supplement, this became a major focus of media stories about the Supplement and was linked to the idea (briefly mentioned above) that the policy might, at any moment, be expanded to other sectors:

*No wonder business suspects a hidden agenda on business rates—what the SNP are doing to supermarkets today they may well be doing to other businesses tomorrow*. —Lewis MacDonald, Labour Party infrastructure spokesman, October 18, 2011^25^

Our data suggest that the alleged potential for the Supplement to create a “worrying precedent” for other forms of business taxation served to widen industry opposition to the measure. Media coverage suggests that, initially, representatives of smaller retailers supported the Supplement. Both the Federation of Small Businesses and the Glasgow Chamber of Commerce were reported to be supportive.^19^, ^26^ However, interviewees from large retailers described how small retailers were encouraged to change their view. For example:

*There was a bit of [support for the Supplement from smaller retail businesses] until it was pointed out [to] some of them: the levy applies today to one group, and the ratable value threshold could quite easily be lowered more particularly if the minister needed some more money in the following year. And that is actually, instead of just, when they were slightly enlightened as to looking at horizon scanning and looking forward a little bit and started to realize actually it might not be such a good thing, they began to change their tune*. —Interviewee from the retail sector

It is clear from the intensity of the industry's response evident in the documents we obtained via Freedom of Information requests, the media coverage of the Supplement, and the comments made in interviews, that large retailers had not expected anything similar to the previous large retailer levy to emerge. The lack of consultation was felt to be particularly damaging in the area of tax policy:

*For tax policy, you should do the evidence beforehand, and you should produce the consultations and explain what the policy is trying to do, and then you should consult tax professionals and other people about how a tax is best designed, and then you should draft some legislation and then consult on that. And that's standard policymaking inside government*. —interviewee from the retail sector

This argument was also apparent in the initial media coverage of the Supplement, with retailers and their trade group representatives expressing their concern over the lack of consultation, especially in the context of Scottish government commitments to working in “partnership” with the business community:

*It's difficult to work in partnership with somebody who keeps bringing surprises with very large bills attached to them…. There is an awful lot of missing detail at the moment. We want to understand what the consultation process is going to be given that there was nothing up until now*. —Jane Bevis, SRC communications director^27^

Both the documentary data and the interviews we undertook indicate that the main actors lobbying against the Supplement were large retailers and the trade groups representing their interests. None of the individuals we interviewed suggested that tobacco or alcohol manufacturers had been active on this issue. However, we did identify evidence that parts of the tobacco industry may have been considering a legal challenge to the policy.

On March 12, 2012, Gallaher Limited (now part of Japan Tobacco International) wrote to Scottish ministers requesting they disclose (1) any “assessment, consideration, or discussion” of the legality of the Supplement in terms of the competence of the Scottish government under the Scotland Act 1998; (2) its effect on competition and/or trade in the European Union; and (3) why the European Commission was not notified about the Supplement.^28^ Scottish ministers responded on March 30, 2012, informing Gallaher that they were withholding the information requested, citing a number of exemptions in Freedom of Information legislation.^28^ Gallaher appealed the government's decision to the Scottish information commissioner but the appeal was ultimately unsuccessful.^28^ None of the individuals we interviewed said they were aware of tobacco industry lobbying about the Supplement or of a potential legal challenge.

### Changes to the Supplement Between Announcement and Implementation

In the period between the initial announcement of the Supplement and the confirmation of its inclusion in the final budget in April 2012, the technical content of the policy and its framing changed in important ways. Specifically, changes were made to (1) the public health rationale, which was progressively de‐emphasized by the Scottish government; (2) the rate at which the tax would be paid, and therefore the predicted revenue from it; and (3) the period of time in which the Supplement would remain in place. In this section, we explain these 3 changes in more detail and comment on the extent to which they are connected to the strong, negative responses of large retailers following the policy's initial announcement.

The presentation by the Scottish government of the rationale for the Supplement changed between the initial announcement and its implementation. This is reflected in emails and letters between the Scottish government and 3 key industry actors—Tesco, CBI Scotland, and the SRC (the latter 2 are the main trade groups that represented large retailers in these debates)—as well as Scottish government minutes of meetings with these and other industry actors, which were obtained through Freedom of Information requests.^29^, ^30^ These documents reveal that, in a meeting on October 17, 2011, with the trade groups in addition to executives from Asda, Morrison's, the Co‐operative Group, and Tesco, Swinney is recorded as stating: “The levy is not an attempt to limit sales or to force retailers to cease sale of these goods, but is to establish a new funding stream to support the long‐term sustainability of public services.”^29^

In a meeting with executives of Tesco on November 10, 2011, Swinney once again emphasized the focus on income generation via the Supplement, stating (according to minutes of that meeting drafted by officials) that “the Scottish government [is] willing to work with Tesco to consider any alternative suggestions for income generation.” This echoed comments he made earlier that day during his visit to a new Shettleston Tesco store and his commitment to meet again with Tesco.^30^

We know from emails between Scottish government officials and Tesco that the latter followed up on this offer and produced a report (which we have not been able to obtain) with a proposal to expand the scope of the tax to include a wider range of large businesses. In a letter from Swinney to David North of Tesco on February 8, 2012 (the morning before the budget speech in which the details of Supplement were confirmed), he thanked the company for the “constructive dialogue” over the Supplement and for the “alternative proposals” that Tesco had suggested. He added: “While I have not pursued these in full, I have given them full consideration and some elements of your suggestions have been taken on board.”^31^

He then confirmed that the details of the Supplement would be confirmed in parliament that afternoon, including “some changes to our original proposals,” stating:

*Having reflected upon your comments and within the constraints of delivering a balanced budget, I have reduced the original amount paid by individual retailers and limited the length of time that the Supplement will apply. This will have the overall effect that the estimated income generated by the Public Health Supplement will reduce by £15 million, to £95 million, over the 3‐year period to 2015. This will be raised by setting a fixed rate Supplement of 9.3 pence in 2012‐13 and 13 pence in 2013 and 2014‐15. […] In addition, I can confirm that the Public Health Supplement will be a temporary measure and will apply for the 3 years of the Spending Review only, from 2012‐13 to 2014‐15.… I hope these measures will go some way to alleviating the concerns expressed by the retail sector about future investment and that we will continue to work together constructively in future*.^31^

To summarize, the documents relating to this period suggest that the “public health” framing of the Supplement, and the spending intentions regarding the revenue to be accrued through this initiative, began to shift as a direct result of the lobbying and scrutiny of large retailers. Policymakers moved away from any clear commitment to pursuing public health goals via the supplement, or hypothecating the revenues for public health, and increasingly emphasized a far less specific connection between the Supplement and agreements with local authorities to increase preventive spending.

## Assessing the Impacts of the Public Health Supplement

### Contribution to Public Sector Spending (and Public Health Spending in Particular)

Given that the rationale for the Supplement was, ultimately, to raise revenue, it is important to note that in this respect the policy was successful: the revenues received by public authorities in Scotland were in line with the (revised, post‐budget) estimates. The measure raised £95.9 million over its 3‐year duration—£0.9 million higher than the amount anticipated when the policy was introduced (author constituency MSP, personal email communication, February 9, 2015). However, the Scottish government has not provided detail of how this money was spent, stating: “The estimated additional income was factored into the total resources available in the Spending Review 2011 and contributed to the preventative spend measures introduced at that time.”^32^

Several interviewees expressed the view that the Supplement was designed in such a way that it was uneconomic for large retailers to avoid—and hence the scale of the revenues to be received could be predicted with accuracy. Indeed, interviewees noted that it was precisely *because* avoiding the Supplement was so difficult that behavior change among retailers (ie, opting not to sell tobacco or alcohol) was unlikely to occur. (It is apparent that, if it does not make economic sense to avoid a tax, in this case by ceasing to sell alcohol or tobacco, then a rational [ie, profit‐maximizing] retailer will not do so.)

Although many of the interviewees claimed that tobacco was not particularly profitable for retailers, they also suggested it was profitable enough to continue selling despite the Supplement and independently of the so‐called footfall effect:

*We modeled it. We looked and said there are [X affected] stores…. [T]he analysis was done on the level of tobacco sales in each store, and even looking at the tobacco sales and the cost it was still worth our while to sell the tobacco, even before you take into account the value of tobacco to us as a driver of footfall. So we looked at it and yeah, it was a no‐brainer to be honest, carry on doing what we were doing*. —Interviewee from a large retailer

This suggests that, if a similar policy were ever intended to encourage retailers to stop selling tobacco (or alcohol), it would need to be set at a substantially higher level.

In terms of the policy's link to preventive public health spending, we could find no evidence of hypothecation. This reflects, in part, a general difficulty of tracing the relationship between specific sources of government revenue and spending on specific activities. However, in this case, it appears that the relationship was particularly indirect. A civil servant in the Scottish Government with experience of working in finance reflected that, despite the supplement's name, the revenue was not exclusively hypothecated to public health spending, and that its name was reflective more of the tax base for the supplement (ie, large retailers of both tobacco and alcohol) than any associated spending.

The picture is further complicated by the fact that the money was raised, not by the Scottish government, but by local authorities in Scotland. Further, local authorities were expected, alongside central government and the National Health Service, to contribute to a £500 million shift to preventive spending, including through 3 so‐called change funds—one focusing on early years, one on reducing reoffending, and one on older people's care.^1^ It could be argued that some of these funds relate to the social determinants of health, and that consequently some of the revenue generated by the Supplement may have been spent on public health–related activities. However, due to the “fungibility” of money^33^ (ie, the fact that any unit of money is substitutable for another), it is difficult to trace the flow between this specific income stream and the change funds: thus, an increase in funding from the Supplement may have been offset by a reduction in other forms of funding by local authorities and central government funding of local authorities. In either case, this would, in effect, mean that there had not been any net increase in the resources available for public health.

### Broader Economic Impacts

The broader economic impacts of the Supplement are even harder to assess, especially in a context in which the large supermarkets were already beginning to change their investment patterns in Scotland. This change was partly a response to challenges from changing economic circumstances, competition from cheaper supermarkets, such as Aldi and Lidl (which were unaffected by the Supplement as neither sell tobacco), and the move to online retail. As a senior policymaker in the Scottish government's health directorate reflected:

*What [large retailers] would claim was, “when we are looking at [a] way to invest in stores, we do the maths, and actually [the Supplement] might not look [like] very much but our margins are so low so we might choose to open a store somewhere elsewhere.[…]” Did I ever fully believe those things? It struck me as unlikely that that margin in itself was likely to be absolutely instrumental to many decisions whether or not to proceed with a store or not…*.

This and other data suggest that, although the Scottish government decided to reduce the level of the Supplement and restrict its implementation to 3 years (as described above), this decision does not appear to have been taken on the basis that Scottish government officials were persuaded by arguments about the negative economic impacts of the Supplement and was more likely a reflection of a desire to recover the broader relationship between the government and large retailers (as discussed in more detail later).

### Public Health Impacts

The Scottish government was asked by the Economy, Energy and Tourism Committee, in a report published on October 9, 2011, to “monitor the impact of the levy on sales of the targeted products, and to report back to Parliament within 18 months on the effectiveness of the measures in terms of (a) public health, (b) revenue generation, and (c) employment in the retail sector.”^34^ Just over a month later, in a letter to the SRC, dated November 15, 2011 (obtained under the Freedom of Information Act), Swinney stated: “Robust mechanisms for measuring effectiveness [of the Supplement] will be developed and implemented by the Scottish government in consultation with its stakeholders.”^35^ We were, however, unable to find any evidence of such reporting. The clerk of the committee confirmed to us that no action had been taken by the committee to follow up on its request (Douglas Wands, email communication, February 9, 2015).

Nonetheless, the media analysis we undertook confirmed that one large supermarket company, Sainsbury's, decided to stop selling tobacco for a period of time in a small number of stores and that the company presented this as a response to the Supplement:

*[T]he impact of the levy introduced by the Scottish government has led us to undertake a review of the sale of tobacco in our Scottish stores. Earlier this year we removed tobacco from 3 of our Scottish supermarkets and 1 convenience store. This trial has been extended to a further 6 supermarkets*. —Sainsbury's spokesperson, November 11, 2012^36^

The extent to which the impetus for Sainsbury's decision to stop selling tobacco in 10 stores really lay with the Supplement is, however, unclear. Industry interviewees expressed skepticism about the stated rationale for this decision. One interviewee from a competitor supermarket company claimed that several of the chosen stores were large, out‐of‐town premises that also had a separate filling station in which tobacco continued to be sold, but that was small enough not to be subject to the Supplement:

*So what [Sainsbury's] did is they stopped selling tobacco in their stores, so their supermarket, but they continued selling tobacco in the kiosk. So when people drove out to the petrol station, filled up their car, they could buy the tobacco there. But because the petrol station and your store are two separate entities in terms of ratable value, so Sainsbury's, people say that's an example of, that was a really good, shows what can be achieved—it achieved nothing*.

Sainsbury's representatives did not agree to be interviewed by us about the Supplement and their response to it.

### The Scottish Government's Credibility, and Relationship, with the Business Community

According to most of our interviewees, one clear impact of the Supplement was damage to the relationship between the Scottish government and the business community (or, at least, the affected retailers):

*It harmed the credibility of the Scottish government with the large retailers.[…] I don't know about other sectors but with food retailers we feel the relationship is not particularly cooperative. Compared to the relationship, say, with Westminster, [which] is far more engaging and, if you think about the public health responsibility deal, there are critics […] [but] what that has achieved is that there is an on‐going dialogue between business and the Department of Health about how best to move things forward.[…] Things are done to retailers in Scotland, whereas in Westminster it's more collaborative.—*Interviewee from a large retailer [interviewee's emphasis]

Although it is hard to provide a definitive account of why, in 2014, the Scottish government decided to announce that the Supplement would not be continued beyond the initial 3‐year period, some interviewees suggested that this decision reflected a perceived need by the Scottish government to improve the relationship with the business community in the context of the 2014 referendum for Scottish independence:

*I would love to tell you it was my fantastic lobbying. I would love to tell you that he'd heard all the arguments, [but] I think the independence referendum had a huge bearing on it, because it did not fit with [the] narrative that in Scotland we'd have a low corporate tax regime. And that was the big message they were playing at that particular juncture, and they went, This doesn't sit, this is going to be a bit of an Achilles’ heel when we come out and talk about independent Scotland cutting corporation tax. What about, “You just increased it £95 million on these other guys”? It didn't sit well, so I think this was a general clearing of the decks ahead of the referendum. So great, huge benefits [of] having the referendum*. —Interviewee from the retail sector

## Discussion

When the Public Health Supplement was first announced in the Spending Review 2011 and Draft Budget 2012‐13, a clear public health rationale for this new tax was advanced: to reduce the economic desirability for large retailers of selling alcohol and tobacco products and increase revenues for preventive health spending. This was also how the Supplement was understood in the public health community, at least initially. In practice, our analysis suggests the Supplement was not designed in such a way as to stimulate behavioral change among retailers, and that the revenues were not hypothecated for health, but used to address a gap in the Scottish government's Spending Review proposals. It could therefore be argued (as many of our interviewees did argue) that the presentation of the Supplement as a “public health” measure was misleading.

After the announcement of the Supplement, large retailers mobilized to lobby against it—through campaigns in the media, Parliament, and direct lobbying of Scottish government ministers and officials. Although some public health–focused NGOs welcomed the measure with brief comments in early media coverage, the absence of genuine health content in the Supplement diminished the involvement of public health advocates, resulting in no sustained attempt to balance the industry's criticism. The lobbying efforts focused on the period between the announcement of the Supplement in September 2011 and its implementation in April 2012, at the end of which major concessions were made by the Scottish government to the industry—including a decision to reduce the tax take from the measure and limit its duration to a 3‐year period. This appears to have been a direct result of what Swinney labeled the “constructive dialogue” initiated via the lobbying campaign.^31^

In this regard, the experience of the Public Health Supplement in Scotland shares some similarities with Denmark's short‐lived “fat tax.” Both measures were met with strong opposition from the commercial sector and in neither case was this opposition countered by support from public health interests.^37^, ^38^ In both cases, the result was a decision not to continue the tax, despite the fact that both could, by some measures, be considered “successful” (the Supplement successfully raised revenue, and the Danish fat tax contributed to reducing the consumption of saturated fat, which was its primary aim^38^). In April 2012, the revenue to be generated by the Supplement was forecast at £95 million. In practice, the amount raised was £95.9 million. The accuracy of the forecast highlights the fact that the tax rate was set at a level that supermarkets would be unlikely to seek to avoid (ie, by ceasing to sell alcohol or tobacco) in the context of the profits earned from such sales. For the same reasons, the Supplement was unlikely to change behavior vis‐à‐vis retailers’ decision to sell alcohol and tobacco. Consistent with this, we did not identify any evidence to suggest that alcohol or tobacco manufacturers were involved in lobbying against the Supplement. However, we did identify evidence to suggest at least one major tobacco manufacturer, Gallaher Limited (now part of Japan Tobacco International), was considering a legal challenge to the policy. This may indicate that a policy that was more directly targeted at trying to achieve retail behavior change with regard to tobacco sales could face legal challenges from this sector.

Given the predictability of the revenue raised through the Supplement, it would have been possible for the Scottish government to develop clear spending proposals, to be funded through this new measure. However, as outlined above, our findings indicate that the Supplement was used to address a gap in the Scottish government's budget rather than to fund any specific set of activities or services. Since these spending plans were already focusing on a shift toward preventive spending, it is plausible that the Supplement helped to support this shift (which is the way one of the Scottish government officials we interviewed framed the outcomes from spending). It is clear, however, that the revenue raised from the Supplement was not meaningfully hypothecated—and indeed it seems likely that there was never any intention to formally hypothecate for health purposes the revenue the Supplement was projected to raise.

In this regard, there are similarities between the experience of the Supplement and many other health taxes that have been initially presented as measures that will raise revenue for spending on particular activities. A systematic review of tobacco industry interests in tobacco taxation has, for example, identified several examples of tobacco tax increases where initial promises of hypothecation for particular purposes were not implemented as planned, with funds often being diverted to other objectives.^10^ This provided tobacco industry interests with an additional argument against hypothecated tax increases.^10^ Although it would not be fair to suggest that Scottish government officials ever made a clear statement of their intent to hypothecate the revenue to public health purposes, the initial framing of the measure implied this would occur, and the failure to do so left the tax with limited support within the public health community.

Our evaluation of the Supplement provides a number of lessons for policy actors who are considering developing, or advocating for, future “health” taxes. The Supplement, as a tax on large retailers selling alcohol and tobacco products, was innovative and, from a health point of view, had some valuable attributes. While it was opposed by large retailers, there does not appear to have been any notable public opposition to the measure. It raised a very predictable revenue stream for the Scottish government, which *could* have been channeled to serve health objectives (even if, in this particular case, this did not occur). On the other hand, while health taxes on particular products are often intended to stimulate behavior change, this was not the intention of the Supplement and, indeed, there does not appear to have been any sustainable behavior change among retailers. Our findings highlight a possible tension between the use of taxes to achieve, on the one hand, revenue objectives (which may or may not be health‐related), and, on the other, public health objectives related to product supply and demand. It is plausible that a similar measure, with a higher tax rate, could be effective in encouraging retailers to stop selling tobacco products, though the predictability of the revenue stream would necessarily diminish. To ascertain at what rate such a measure would need to be set would require detailed scenario modeling.

In addition, it is possible that the extent of behavior change would have been greater had the measure been extended to smaller retailers—though, in this case, it is also possible that the measure would have faced stronger, and broader, opposition. (It is perhaps worth noting that, at the same time Scotland implemented the Public Health Supplement, the Northern Ireland Executive chose to introduce a tax on larger retailers, without any “health” focus, and positioned this as part of a smaller business agenda.^39^) It also seems feasible that, as has been suggested with health taxes on particular products, such a measure could be supported by subsidies to “healthy businesses,” helping to reduce the likely opposition to such a scheme. For example, businesses that volunteered to stop selling tobacco and/or alcohol products could be offered a “healthy business” subsidy that would be funded by the tax on retailers that continued to sell these products. Again, some detailed scenario modeling of possible options would be required to examine this idea in more detail. Such a measure could, in principle, be self‐financing if the revenue stream from the levy was hypothecated, in whole or in part, to that purpose. At the very least, Scotland's experience with the Public Health Supplement provides a starting point for developing proposals for policies intended to limit the availability of tobacco and alcohol products (proposals that are likely to be of particular interest to those working in contexts in which there appears to be an existing over‐supply, as is the case for Scotland in general, and deprived areas of Scotland in particular.^40^)

Overall, our findings suggest that it would be worth considering a future health tax of a similar design and level to the Supplement if the objective is to raise revenue for health spending. If the objective is to change retail behavior (eg, to encourage retailers to stop selling tobacco products), then further research is required to ascertain the likely level at which change would occur and also to better understand whether retailer concerns about such a policy might be diminished by linking the revenue to a “healthy retailer” subsidy.
